# Protective antioxidative effects of caffeic acid phenethyl ester (CAPE) in the thyroid and the liver are similar to those caused by melatonin

**DOI:** 10.1186/1756-6614-7-5

**Published:** 2014-06-05

**Authors:** Agnieszka Kokoszko-Bilska, Jan Stepniak, Andrzej Lewinski, Malgorzata Karbownik-Lewinska

**Affiliations:** 1 Department of Oncological Endocrinology, Medical University of Lodz, 7/9 Zeligowski St, Lodz 90-752, Poland; 2 Department of Endocrinology and Metabolic Diseases, Medical University of Lodz, 281/289 Rzgowska St, Lodz 93-338, Poland; 3 Polish Mother’s Memorial Hospital – Research Institute, 281/289, Rzgowska St, Lodz 93-338, Poland

**Keywords:** Caffeic acid phenethyl ester (CAPE), Melatonin, Oxidative stress, Lipid peroxidation (LPO), Antioxidant, Thyroid, Liver

## Abstract

**Background:**

Whereas oxidative reactions occur in all tissues and organs, the thyroid constitutes such an organ, in which oxidative processes are indispensable for physiological functions. In turn, numerous metabolic reactions occurring in the liver create favourable conditions for huge oxidative stress. Melatonin is a well-known antioxidant with protective effects against oxidative damage perfectly documented in many tissues, the thyroid and the liver included. Caffeic acid phenethyl ester (CAPE), a component of honeybee propolis, has been suggested to be also an effective antioxidant.

The aim of the study was to evaluate the effects of CAPE on Fenton reaction-induced oxidative damage to membrane lipids (lipid peroxidation, LPO) in porcine thyroid and liver, and to compare the results with protective effects of melatonin.

**Methods:**

Thyroid and liver homogenates were incubated in the presence of CAPE (500; 100; 50; 10; 5.0; 1.0 μM) or melatonin (500; 100; 50; 10; 5.0; 1.0 μM), without or with addition of FeSO_4_ (30 μM) + H_2_O_2_ (0.5 mM).

The level of lipid peroxidation was measured spectrophotometrically and expressed as the amount of MDA + 4-HDA (nmol) per mg of protein.

**Results:**

Whereas CAPE decreased the basal LPO in a concentration-dependent manner in both tissues, melatonin did not change the basal LPO level. When antioxidants were used together with Fenton reaction substrates, they prevented – in a concentration-dependent manner and to a similar extent – experimentally-induced LPO in both tissues.

**Conclusions:**

Protective antioxidative effects of CAPE in the thyroid and the liver are similar to those caused by melatonin. CAPE constitutes a promising agent in terms of its application in experimental and, possibly, clinical studies.

## Background

Whereas oxidative reactions occur in all tissues and organs, the thyroid gland constitutes such an organ, in which oxidative processes are indispensable for physiological functions, thyroid hormone biosynthesis included. It is estimated that huge amount of reactive oxygen species (ROS), especially of hydrogen peroxide (H_2_O_2_), are produced in the thyroid under physiological conditions, justifying the statement that the thyroid gland is an organ of “oxidative nature” [1,2]. However, with additional oxidative abuse caused by exogenous or endogenous prooxidants, increased oxidative damage to all major cellular components, such as lipids, proteins and DNA, may occur, leading to different thyroid diseases, cancer included [1-4].

In turn, numerous metabolic reactions occurring in the liver create favourable conditions for huge oxidative stress. ROS and reactive nitrogen species (RNS), generated during these processes, are very important factors, not only in the physiology, but also in the pathophysiology of the hepatocyte [5,6]. The significant role of increased oxidative damage in the pathogenesis of different liver diseases, such as viral hepatitis, alcoholic hepatitis, hemochromatosis, drug-induced liver injury, hepatic insulin resistance, non-alcoholic fatty liver disease or hepatocellular cancer, has been demonstrated in many experimental and clinical studies [5-8].

Due to potential huge oxidative stress in both the thyroid and the liver, effective protective mechanisms should have been developed. In these organs, the antioxidative defence systems are very extensive and comprise both enzymatic and non-enzymatic antioxidants [2,6]. On the other hand, the use of various exogenous antioxidants in both tissues – under condition of increased oxidative stress – has been shown to be beneficial in experimental (*in vitro* or *in vivo*) studies [2,5,9,10]. Unfortunately, the applicability of different antioxidants in human thyroid or liver diseases has not been yet clearly confirmed in clinical studies and still no effective antioxidative medication is available for widespread use [6-8,10]. Moreover, some antioxidants, such as ascorbic acid (vitamin C), present a dual nature, acting in different redox environment as an antioxidant or prooxidant [11].

Melatonin (N-acetyl-5-methoxytryptamine), produced in the pineal gland, as well as in numerous other tissues and organs, is a very well-known antioxidant and free radical scavenger, with protective effects against oxidative damage perfectly documented in many tissues, the thyroid and the liver included [9,12-16].

Caffeic acid phenethyl ester (CAPE), an active component of honeybee propolis extract, is structurally related to flavonoids and has been used as a traditional medicine for many years [17-19]. It is known to possess numerous biological activities including anti-inflammatory, antiviral and immunomodulatory properties [17-19]. In experimental studies, CAPE has been shown to inhibit the growth and metastasis of different types of tumor, to protect tissues from reperfusion injury in various ischemia-reperfusion models, and to suppress inflammation in a variety of tissues [20]. It has been suggested to be also an effective antioxidant [21]. CAPE might be even used as a protective agent against chemotherapy-induced and radiotherapy-induced toxicity [17].

The aim of the study was to evaluate the effects of CAPE on experimentally-induced oxidative damage to membrane lipids (lipid peroxidation, LPO) in porcine thyroid and liver homogenates, and to compare the results with protective effects of melatonin. Substrates of Fenton reaction (Fe^2+^+H_2_O_2_ → Fe^3+^+^•^OH + OH^-^) – the most basic reaction of oxidative stress – were used to induce oxidative damage to membrane lipids.

## Methods

The procedures, used in the study, were approved by the Ethics Committee of the Medical University of Lodz, Poland.

### Chemicals

Caffeic acid phenethyl ester (CAPE), melatonin, ferrous sulfate (FeSO_4_) and hydrogen peroxide (H_2_O_2_) were purchased from Sigma-Aldrich (St. Louis, MO). The ALDetect™ Lipid Peroxidation Assay Kit for LPO was obtained from Enzo Life Science (Farmingdale, NY). All the used chemicals were of analytical grade and came from commercial sources.

### Animals

Porcine thyroids and livers were collected from twenty one (21) animals at a slaughter-house, frozen on solid CO_2_ and stored at -80°C until assay. Three independent experiments were performed. Therefore, three tissue pools were prepared, with seven (7) thyroid glands used for each homogenate pool.

### Incubation of thyroid and liver homogenates

Thyroid and liver tissue were homogenized in ice cold 50 mM Tris–HCl buffer (pH = 7.4) (10%, w/v), and then incubated for 30 min at 37°C in the presence of examined substances.

Tissue homogenates were incubated in the presence of:

Experiment I: CAPE (500; 100; 50; 10; 5.0; 1.0 μM) alone (to check its effect on the basal LPO) or with addition of Fenton reaction substrates [FeSO_4_ (30 μM) + H_2_O_2_ (0.5 mM)].

Experiment II: melatonin (500; 100; 50; 10; 5.0; 1.0 μM) alone (to check its effect on the basal LPO) or with addition of Fenton reaction substrates [FeSO_4_ (30 μM) + H_2_O_2_ (0.5 mM)].

The concentrations of FeSO_4_ (30 μM) and H_2_O_2_ (0.5 mM) were selected from our previous study [12].

The reactions were stopped by cooling the samples on ice. Each experiment was run in duplicate and repeated three times.

### Measurement of lipid peroxidation products

Concentrations of malondialdehyde + 4-hydroxyalkenals (MDA + 4-HDA), as an index of LPO, were measured in tissue homogenates.

The homogenates were centrifuged at 3000 x g for 10 min at 4°C. The supernatant was mixed with 650 μl of a methanol:acetonitrile (1:3, v/v) solution, containing a chromogenic reagent, N-methyl-2-phenylindole, and vortexed. After addition of 150 μl of methanesulfonic acid (15.4 M), the incubation was carried out at 45°C for 40 min. The reaction between MDA + 4-HDA and N-methyl-2-phenylindole yielded a chromophore, which was spectrophotometrically measured at the absorbance of 586 nm, using a solution of 4-hydroxynonenal (10 mM) as standard.

The level of LPO was expressed as the amount of MDA + 4-HDA (nmol) per mg of protein.

### Protein measurement

Protein was measured, using Bradford’s method [22], with bovine albumin as the standard.

### Statistical analysis

Results are expressed as means ± SE. Data were statistically analyzed, using a one-way analysis of variance (ANOVA), followed by the Student-Neuman-Keuls’ test. The level of p < 0.05 was accepted as statistically significant.

Statistica for Windows 10.0 software was used for the statistical analysis.

## Results

**Figure 1:**
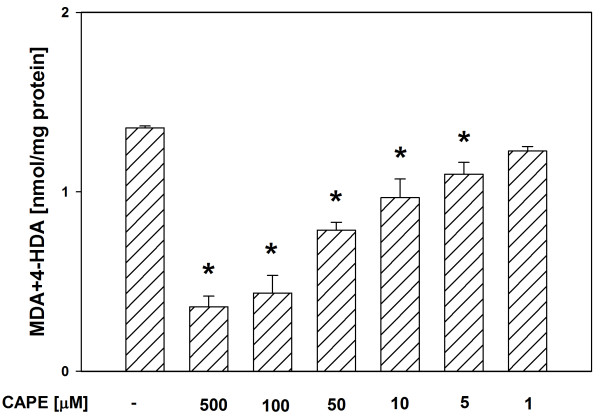
**Concentrations of MDA + 4-HDA in porcine thyroid homogenates incubated for 30 min in presence of CAPE (500, 100, 50, 10, 5, 1 μM).** Bars represent means ± SEM of three independent experiments run in duplicates. *p < 0.05 versus Controls.

**Figure 2:**
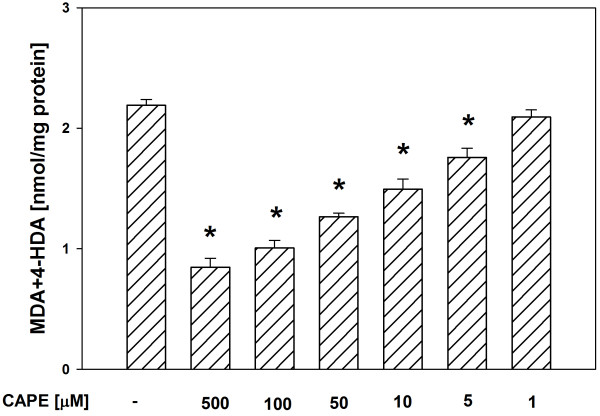
**Concentrations of MDA + 4-HDA in porcine liver homogenates incubated for 30 min in presence of CAPE (500, 100, 50, 10, 5, 1 μM).** Bars represent means ± SEM of three independent experiments run in duplicates. *p < 0.05 versus Controls.

**Figure 3:**
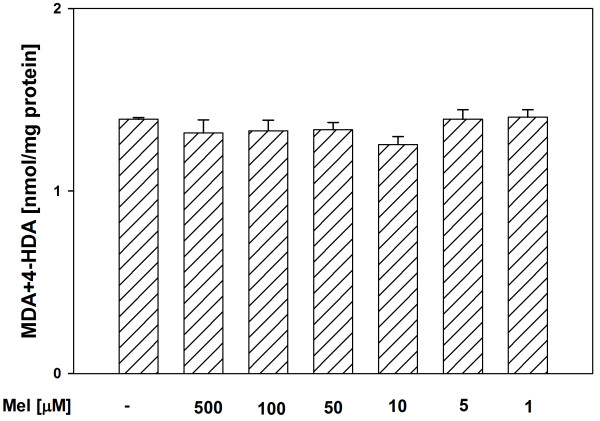
**Concentrations of MDA + 4-HDA in porcine thyroid homogenates incubated for 30 min in presence of melatonin (500, 100, 50, 10, 5, 1 μM).** Bars represent means ± SEM of three independent experiments run in duplicates.

**Figure 4:**
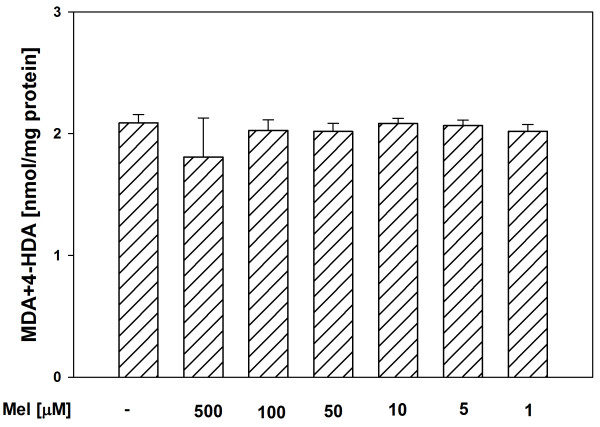
**Concentrations of MDA + 4-HDA in porcine liver homogenates incubated for 30 min in presence of melatonin (500, 100, 50, 10, 5, 1 μM).** Bars represent means ± SEM of three independent experiments run in duplicates.

In both tissue homogenates, CAPE (used in concentrations of 500, 100, 50, 10 and 5 μM) decreased the basal LPO in a concentration-dependent manner (Figures 1, 2), while melatonin (in all the used concentrations) did not change the basal LPO level (Figures 3, 4).

**Figure 5:**
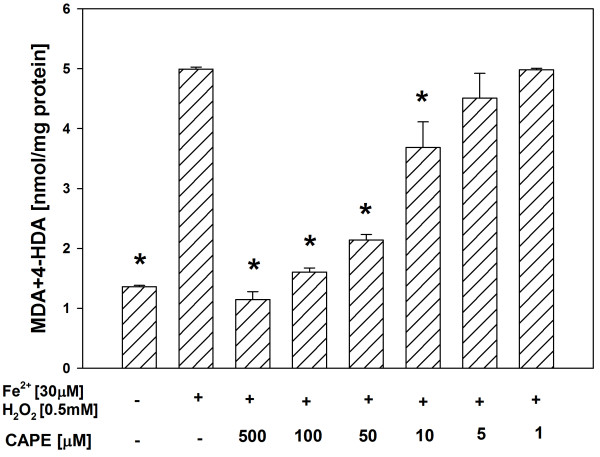
**Concentrations of MDA + 4-HDA in porcine thyroid homogenates incubated for 30 min in presence of FeSO**_**4 **_**(30 μM) + H**_**2**_**O**_**2 **_**(0.5 mM), or FeSO**_**4 **_**(30 μM) + H**_**2**_**O**_**2 **_**(0.5 mM) + CAPE (500, 100, 50, 10, 5, 1 μM).** Bars represent means ± SEM of three independent experiments run in duplicates. *p < 0.05 versus Fe^2+^ (30 μM) + H_2_O_2_ (0.5 mM).

**Figure 6:**
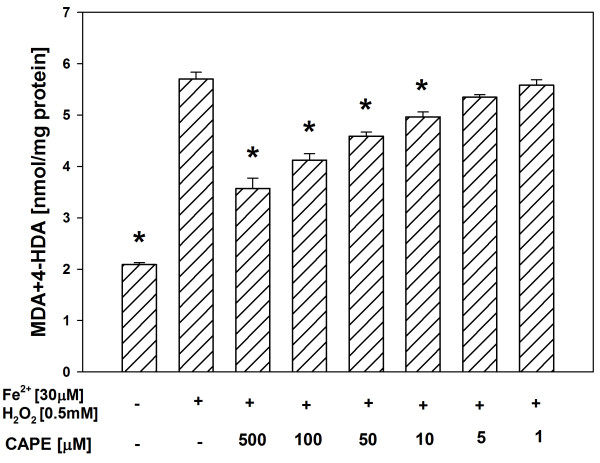
**Concentrations of MDA + 4-HDA in porcine liver homogenates incubated for 30 min in presence of FeSO**_**4 **_**(30 μM) + H**_**2**_**O**_**2 **_**(0.5 mM), or FeSO**_**4 **_**(30 μM) + H**_**2**_**O**_**2 **_**(0.5 mM) + CAPE (500, 100, 50, 10, 5, 1 μM).** Bars represent means ± SEM of three independent experiments run in duplicates. *p < 0.05 versus Fe^2+^ (30 μM) + H_2_O_2_ (0.5 mM).

**Figure 7:**
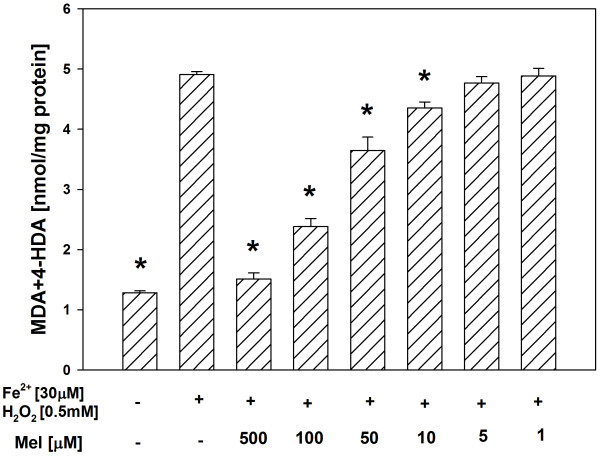
**Concentrations of MDA + 4-HDA in porcine thyroid homogenates incubated for 30 min in presence of FeSO**_**4 **_**(30 μM) + H**_**2**_**O**_**2 **_**(0.5 mM), or FeSO**_**4 **_**(30 μM) + H**_**2**_**O**_**2 **_**(0.5 mM) + melatonin (500, 100, 50, 10, 5, 1 μM).** Bars represent means ± SEM of three independent experiments run in duplicates. *p < 0.05 versus Fe^2+^ (30 μM) + H_2_O_2_ (0.5 mM).

**Figure 8:**
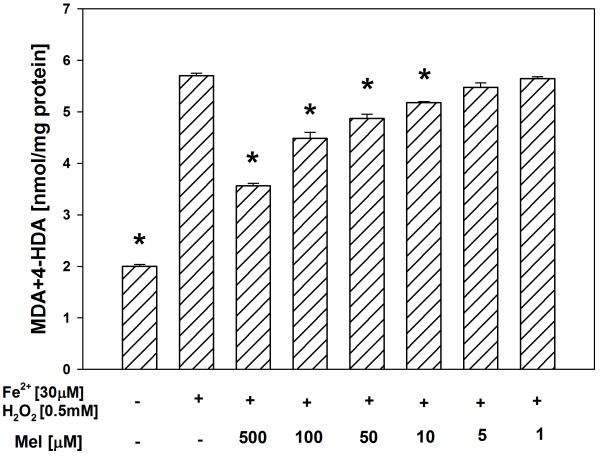
**Concentrations of MDA + 4-HDA in porcine liver homogenates incubated for 30 min in presence of FeSO**_**4 **_**(30 μM) + H**_**2**_**O**_**2 **_**(0.5 mM), or FeSO**_**4 **_**(30 μM) + H**_**2**_**O**_**2 **_**(0.5 mM) + melatonin (500, 100, 50, 10, 5, 1 μM).** Bars represent means ± SEM of three independent experiments run in duplicates. *p < 0.05 versus Fe^2+^ (30 μM) + H_2_O_2_ (0.5 mM).

In all the experiments, Fenton reaction substrates (FeSO_4_ and H_2_O_2_), added into the incubation medium, increased the level of LPO in both the thyroid (Figures 5, 6) and the liver (Figures 7, 8).

Antioxidants, i.e. CAPE or melatonin, prevented – in a concentration-dependent manner – experimentally-induced LPO in both the thyroid (Figures 5, 6) and the liver (Figures 7, 8), when used in the same range of concentrations (i.e. 500, 100, 50 or 10 μM).

## Discussion

According to our knowledge, the present study has been the first attempt to evaluate the effects of CAPE on the basal and experimentally-induced LPO in the thyroid and liver homogenates under *in vitro* conditions. Also for the first time, we compared the antioxidative activities of CAPE and melatonin in the thyroid.

The selection of the thyroid and the liver for such a study is justified by several factors. The thyroid gland is an organ in which ROS are widely involved in its function and even are indispensable for physiological processes [1,2]. Hydrogen peroxide acts as an electron acceptor at each step of thyroid hormone biosynthesis and is essential for activity of thyroperoxidase (TPO) – the key enzyme in this process [1,2]. Also, the activity of antioxidative enzymes [superoxide dismutase (SOD), glutathione (GSH) peroxidase (GSH-Px) and catalase (CAT)] and antioxidative proteins (such as α- and γ-tocopherols, coenzyme Q, ascorbic acid and peroxiredoxins) has been well documented in the thyroid [1,2]. The liver plays an important role in detoxifying processes. It is also one of the organs with a higher oxygen consumption rate and therefore constitutively presents a greater expression of antioxidative enzymes than those with lower oxygen consumption [5]. Hepatocytes contain a variety of antioxidant enzymatic systems (SOD, GSH-Px, CAT), so they are able to reduce the amount of oxygen free radicals generated during cellular activity [6].

Consistently with our observations, many previous studies, conducted under *in vivo* conditions, have shown that CAPE exhibits antioxidant properties in different tissues. For example, CAPE, injected intraperitoneally (*i.p*.) to 10–12 weeks old male Wistar albino rats (10 mg/kg body mass; 10-days administration), revealed protective effects on the aflatoxin B1-induced hepatoxicity by modulating free radical production: it decreased the total oxidant capacity (TOC) and glutathione S-transferase activity [23]. In another study, CAPE, given *i.p*. to 12 weeks old female Wistar albino rats (10 μmol/kg body mass; 6-days administration), prevented cisplatin-induced oxidative changes in the liver by reducing ROS production and increasing antioxidative enzyme activities (such as SOD, GSH-Px and CAT) [24]. Caffeic acid phenethyl ester also revealed the protective effects against liver damage induced by cigarette smoking [25], amikacin-induced nephrotoxicity [26] and methotrexate-induced hepatorenal oxidative injury [27].

Because iron is present in TPO and H_2_O_2_ is indispensable for TPO activity, the thyroid gland may be exposed – under certain conditions – to excessive amounts of either Fe^2+^ or H_2_O_2_, or both, creating favorable conditions for additional Fenton reaction and, consequently, oxidative damage. Therefore, the observation – made by us in the present study – of the protective antioxidative effects of CAPE on Fenton reaction-induced LPO in the thyroid, seems to be of a great importance, especially that antioxidative properties of CAPE are mainly due to its free radical scavenging activity and ferrous ions chelating capacity [25]. Caffeic acid and its derivatives, such as CAPE, may chelate the ferrous ions with hydroxyl groups and then stabilizing the oxidized form of the metal ion [28].

In our study, CAPE revealed similar protective antioxidative effects to those caused by melatonin. These findings are in agreement with the results of previous studies performed under *in vivo* conditions. In 8-week old male Spraque-Dawley rats, melatonin (100 μg/kg body mass daily, for 60 days) or CAPE (10 μmol/kg body mass daily, for 60 days) preinjections reduced – to a similar extent – retinal oxidative stress caused by a long-term exposure to electromagnetic radiation (900 MHz) emitting by mobile phone [29]. In other study, melatonin and CAPE were efficient in delaying age-related cellular damage in cardiovascular system [30]. The aim of this study was to evaluate the effect of chronic melatonin (5 mg/kg body mass; injected daily for 95 days) and CAPE (15 mg/kg body mass; injected daily for 95 days) administration on the ultrastructural features, LPO levels, and enzymatic and nonenzymatic antioxidants in the heart and aorta of aged male Sprague–Dawley rats. Melatonin and CAPE administration significantly reduced the levels of MDA (as an index of LPO), increased the levels of the antioxidative enzymes and reduced age-related histological changes (such as nuclear irregularity, mitochondrial degeneration myofilament disorganization and disruption) in both heart and thoracic aorta of aged rats [30].

Of a great importance is the fact, that in the present study, in both tissue homogenates, CAPE decreased the basal LPO in a concentration-dependent manner, while melatonin (in all the used concentrations) did not change the basal LPO level. It is well known (from many *in vivo* and *in vitro* studies) that melatonin is a perfect antioxidant because of its effective protective action against damaging effects of different prooxidants and the fact that under basal conditions it does not change the level of oxidative damage to macromolecules [12,13,31,32]. The observation – made by us in the present study – of the decreasing effect of CAPE on the basal LPO level, was unexpected and also undesirable. This finding may indicate that CAPE is not as perfect antioxidant as it seemed based on the results of earlier studies. According to our knowledge, only in two studies the effect of CAPE on the basal LPO was evaluated under *in vivo* conditions [24,33]. In the quoted studies, CAPE (injected for 6 days at a dose of 10 μmol/kg body mass [24] or for 3 days at the same dose [33]) did not change the basal LPO level [24,33]. This apparent disagreement between the above quoted studies and the present results may be explained by the fact that the *in vitro* effects can not be directly extrapolated to the *in vivo* conditions.

## Conclusions

In conclusion, protective antioxidative effects of CAPE in the thyroid and the liver are similar to those caused by melatonin. CAPE constitutes a promising agent in terms of its application in experimental and, possibly, clinical studies.

## Abbreviations

CAPE: Caffeic acid phenethyl ester; CAT: Catalase; FeSO_4_: Ferrous sulfate; GSH: Glutathione; GSH-Px: Glutathione peroxidase; H_2_O_2_: Hydrogen peroxide; LPO: Lipid peroxidation; RNS: Reactive nitrogen species; ROS: Reactive oxygen species; SOD: Superoxide dismutase; TOC: Total oxidant capacity; TPO: Thyroperoxidase.

## Competing interests

The authors have declared that there is no conflict of interest.

## Authors’ contribution

AK-B and MK-L designed the study and prepared the final version of the manuscript. MK-L supervised the conduction of the study. AK-B and JS carried out the experiments and performed the statistical evaluation. AK-B prepared the draft of the manuscript. AL revised the final version of the manuscript. All authors read and approved the final manuscript.
